# The Association between Green and Black Tea Consumption on Successful Aging: A Combined Analysis of the ATTICA and MEDiterranean ISlands (MEDIS) Epidemiological Studies

**DOI:** 10.3390/molecules24101862

**Published:** 2019-05-15

**Authors:** Nenad Naumovski, Alexandra Foscolou, Nathan M. D’Cunha, Stefanos Tyrovolas, Christina Chrysohoou, Labros S. Sidossis, Loukianos Rallidis, Antonia-Leda Matalas, Evangelos Polychronopoulos, Christos Pitsavos, Demosthenes Panagiotakos

**Affiliations:** 1 Faculty of Health, University of Canberra, 2617 Canberra, Australia; Nenad.Naumovski@canberra.edu.au (N.N.); Nathan.D’Cunha@canberra.edu.au (N.M.D.); 2 Collaborative Research in Bioactives and Biomarkers (CRIBB) Group, University of Canberra, 2617 Bruce, Australia; 3 Department of Nutrition and Dietetics, School of Health Science and Education, Harokopio University, 176 76 Athens, Greece; alexandra.foscolou@gmail.com (A.F.); s.tyrovolas@pssjd.org (S.T.); lss133@rci.rutgers.edu (L.S.S.); amatala@hua.gr (A.-L.M.); evpol@hua.gr (E.P.); 4 Parc Sanitari Sant Joan de Déu, Fundació Sant Joan de Déu, CIBERSAM, Universitat de Barcelona, 08007 Barcelona, Spain; 5 First Cardiology Clinic, School of Medicine, University of Athens, 106 79 Athens, Greece; chrysohoou@usa.net (C.C.); cpitsavo@med.uoa.gr (C.P.); 6 Department of Kinesiology and Health, School of Arts and Sciences, Rutgers University, NJ 08901, USA; 7 Second Cardiology Clinic, School of Medicine, University of Athens, 106 79 Athens, Greece; lrallidis@gmail.com

**Keywords:** green tea, EGCG, tea consumption, successful ageing index, cardiovascular disease

## Abstract

Tea is one of the most-widely consumed beverages in the world with a number of different beneficial health effects, mainly ascribed to the polyphenolic content of the tea catechins. The aim of this study was to examine the consumption of green, black, or no tea, in relation to the previously validated successful ageing index (SAI; higher values “healthier” ageing) in a combined analysis of adults aged >50 years old from the ATTICA (*n* = 1128 adults from Athens, Greece metropolitan area) and the MEDiterranean Islands Study (MEDIS) (*n* = 2221 adults from various Greek island and Mani) studies. After adjusting for age, sex, smoking, and coffee consumption, green tea was positively associated with SAI (b ± SE: 0.225 ± 0.055, *p <* 0.001), while black tea was negatively associated with SAI (unstandardized b coefficient ± Standard error: −0.807 ± 0.054, *p* < 0.001). Green tea (vs black tea) consumption, had higher odds of a SAI of over 3.58 out of 10 (OR: 1.77, 95% CI: 1.38–2.28). Green tea consumption was also associated with higher levels of physical activity (*p <* 0.001) and reduced likelihood of hypertension (*p* = 0.006) compared with black tea. Two possible mechanisms are that green tea possesses high levels of catechins such as (−)-epigallocatechin 3-gallate and l-theanine compared with black tea. Therefore, the present analysis supports both the role of green tea constituents in successful ageing, as well as its role as an important component of an overall healthy diet in adults aged 50 years and over from these two epidemiological studies.

## 1. Introduction

Tea is one of the most widely consumed beverages worldwide, and global consumption ranks second only to water, well ahead of coffee, beer, wine and carbonated soft drinks [1,2]. Traditionally, the Chinese and Japanese people have believed that drinking tea promotes optimal health and longevity [3,4]. Therefore, it is not surprising that the health-promoting effects of tea have been intensively investigated over recent decades [3,5,6]. Prior to consumption, tea is generally processed and relatively recent research has classified tea originating from the plant *Camellia sinensis* into seven types based on the type of processing as; ‘green’ (unfermented), ‘yellow’, ‘white’, ‘oolong’ (partially fermented), ‘black’ (completely fermented), ‘aged pu-erh’ (drastically fermented and aged) and ‘ripened pu-erh’ tea [7,8]. The global consumption of tea type varies, with black tea the most popular and favored in Western countries [9], green tea favored in Oriental countries, and oolong and pu-erh tea predominately consumed in China [3,10,11]. 

The manufacture of black tea involves the oxidative polymerization of the monomeric flavan-3-ols by the enzyme polyphenol oxidase, leading to the formation of bisflavanols, theaflavins, thearubigins and other oligomers. In contrast, during the production of green tea, freshly harvested leaves are quickly steamed or hot air-dried to inhibit the oxidizing enzyme, polyphenol oxidase, which prevents fermentation of the tea, yielding a dry and stable product. The blanching of leaves caused by this exposure of tea to hot steam or air is also responsible for the production of its pronounced green color [1,6]. The composition of green tea varies with the tea plant variety, harvest season, the position of the plucked leaves, horticultural practices, climate and geographical position [11]. However, all green teas are rich in polyphenols, including flavadiols, flavonoids and phenolic acids. The beneficial health effects of green tea are primarily ascribed to the biologically active polyphenolic components, especially the monomeric flavan-3-ols and the catechins [3,10,11,12]. 

Catechins are the most prominent and biologically active compounds in green tea, almost all of which are extracted during the tea brewing process. These compounds also provide a slightly astringent and bitter taste to green tea infusions with one cup (2.5 g/200 mL) containing approximately 150–200 mg of catechins in total [6]. The content of catechins in green tea can reach up to 30% of the tea leaves’ dry weight [13] with (−)-epigallocatechin 3-gallate (EGCG) being the most predominant, accounting for up to 60% of the total catechin content [14]. Nevertheless, the (−)-epigallocatechin (EGC) was also reported to be one of the major catechins found in several green tea samples [15,16], making EGCG and EGC, undoubtedly the major catechins present in green tea infusions. However, oolong, black and pu-erh teas are allowed to be fermented and oxidized, lowering their catechin content due to their conversion to theaflavins and thearubigins [3]. 

**Figure 1:**
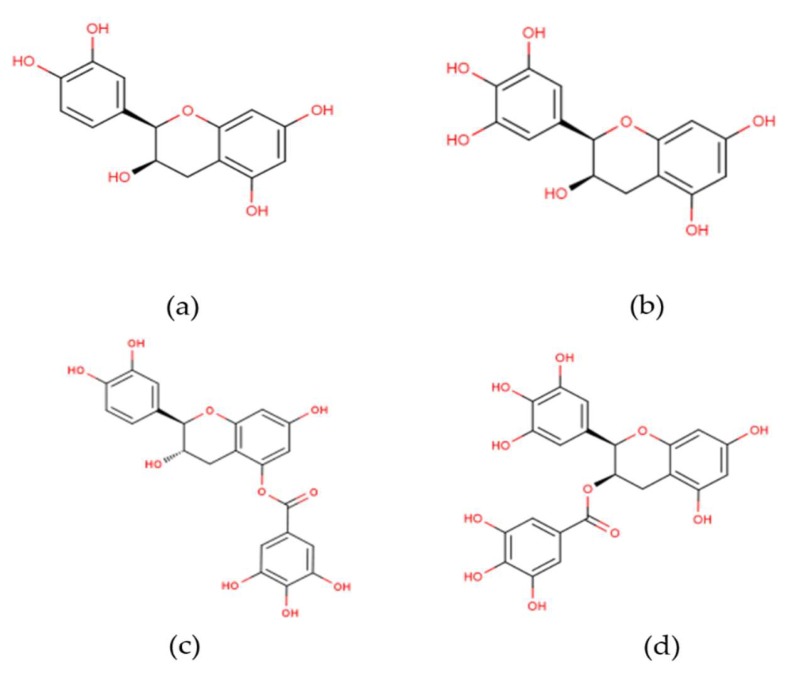
Chemical structures of the four main green tea catechins (**a**) Epicatechin; (**b**) Epigallocatechin; (**c**) Epicatechin-3-gallate; (**d**) Epigallocatechin-3-gallate. Adapted from [6,18].

Green tea polyphenols are predominately comprised of four major catechins; in decreasing order of the amount present in tea, are: EGCG; (−)-epigallocatechin (EGC); (−)-epicatechin 3-gallate (ECG) and (−)-epicatechin (EC) [6,11]. The structure of these four catechins (Figure 1) is characterized by multiple hydroxyl groups on two benzene rings. The gallocatechins (EGC and EGCG) have an extra hydroxyl group in the 5′ position of the B ring while the catechin gallates (ECG and EGCG) have an extra benzene ring with three hydroxyl groups [17].

There is increasing evidence from epidemiological, clinical and experimental studies which suggest that the tea polyphenols (EGCG in particular) have various biological activities such as anti-fungal [19], anti-inflammatory [20,21] and antioxidative [22] properties. Habitual green tea consumption is associated with protection against several types of cancer [23] and lower all-cause mortality [24], while total consumption of tea has shown beneficial changes in psycho-cardiological outcomes, such as reduced risk of cardiovascular disease [25] and type 2 diabetes [26], lower blood pressure, reduced risk of developing hypertension in older adulthood [27], and decreased risk of cognitive impairment [28]. Black tea consumption is also associated with decreased risk of the development of cardiovascular disease in a ‘normal’ population [29]. Interestingly, two relatively recent meta-analyses of randomized controlled trials of black tea consumption on concentrations of serum cholesterols reported conflicting results with one showing no beneficial effects [30], and the other finding lower low density lipoprotein cholesterol (LDL-C) [31]. Nevertheless, higher tea consumption is considered part of a healthy lifestyle that may benefit health through various ways [25,29]. 

Aging is a continuous process associated with a higher likelihood of chronic disease and risk of increased morbidity and mortality [32]. The term successful aging incorporates many aspects of healthy living including quality of life, well-being, physical health, and the maintenance of cognitive function [33]. The role of dietary patterns on successful ageing is becoming better understood, with a current focus on the Mediterranean diet and its predominant constituents which include fruits and vegetables [34], and olive oil [35]. However, the potential health amplifying effects of tea consumption in the context of a Mediterranean style dietary pattern are unclear. Therefore, this study aimed to examine the association of tea consumption (green, black and no tea) on successful aging in people aged 50 years and over, living in Greece.

## 2. Results

In Table 1 some of the socio-demographic data, lifestyle and clinical characteristics of the ATTICA and MEDIS participants, categorized by type of tea consumption, are presented and used for the analyses. From the total of 1195 participants consuming tea, participants with green tea consumption were more frequently male (*p* = 0.026) and physically active (*p* < 0.001), while they were less likely to have hypertension (*p* = 0.006) and more likely to have a higher level of the successful aging index (SAI) (*p* < 0.001), as compared to those consuming black tea. No statistically significant differences were observed as regards cardiometabolic risk factors, adherence to the Mediterranean diet and smoking habits (all *p*’s > 0.05).

A significant correlation was observed between the type of tea consumed and SAI score (Spearman rho = −0.232, *p* < 0.001); thus, to further test the research hypothesis, multiple linear regression analysis was then applied (Table 2). After adjustments (Table 2) for age, sex, smoking habits and coffee intake, drinking, only black tea was negatively associated with SAI levels (b ± SE: −0.727 ± 0.055, *p* < 0.001), whereas drinking green tea vs black tea was positively associated with SAI levels (b ± SE: 0.503 ± 0.078, *p <* 0.001). Additionally, drinking exclusively green tea was positively associated with SAI levels (b ± SE: 0.225 ± 0.055, *p* < 0.001) compared to not drinking at all tea (control group), whereas drinking black tea vs no drinking at all tea was inversely associated with SAI levels (*p* < 0.001). Moreover, drinking green tea but no black or not drinking at all tea was not significantly associated with SAI levels (*p =* 0.722). Black tea consumption compared with no tea at all was negatively associated with SAI levels (b ± SE: −0.807 ± 0.054, *p* < 0.001). Therefore, it is concluded that the consumption of green tea among individuals aged > 50 years is associated with more favorable SAI levels (Table 2).

In Table 3**,** results are presented from logistic regression models that evaluated the association between the type of tea and the SAI level. Regarding the overall sample, those drinking green tea had 1.77 times higher odds (95%CI, 1.38–2.28) for having SAI over 3.58 compared to those drinking black tea, while those drinking green tea had 1.41 times higher odds (95% CI, 1.15–1.73) for having SAI over the median compared to those drinking black tea or the control group. Moreover, regarding those drinking black tea, the odds of having high SAI levels were 38% less than those drinking green or the control group. Similar logistic regression models based on gender showed that females and males drinking green tea had 1.75 and 1.79 times, respectively, higher odds (95%CI, 1.22–2.52 and 1.26–2.53 respectively) for having SAI over 3.58 compared to those females and males drinking black tea. Accordingly, those females and males drinking green tea had 1.36 and 1.50 times, respectively, higher odds (95% CI, 1.01–1.84 and 1.12–2.01 respectively) for having SAI over the median compared to those females and males drinking black tea or the control group. Finally, regarding those females and males drinking black tea, the odds of having high SAI levels were 38% and 33% less than those females and males drinking green or the control group, respectively.

## 3. Discussion

The present pooled analysis of two large-scale population-based studies conducted in Greece assessed the association between green and black tea consumption on successful aging in older people. The main findings demonstrated that the consumption of green tea was linked to a higher likelihood of successful aging based on the SAI compared with consumption of black tea following adjustment for potential confounding factors including age, sex, smoking, and coffee consumption. Interestingly, black tea consumption was negatively associated with successful aging compared with both green tea consumption and no tea consumption. Therefore, green tea, but not black tea, may be associated with enhanced healthy aging in older people in this population living in the Mediterranean region. 

While both green tea and black tea are widely considered as healthy beverages, only green tea was associated with a higher SAI while in contrast, black tea was associated with a lower SAI. No differences in average daily consumption of the number of participants consuming green and black tea were observed. Although both green and black tea are high in antioxidants such as polyphenols and flavonoids, green tea possesses high amounts of catechins such as EGCG. In human trials, EGCG has been shown to promote fat oxidation and increase postprandial thermogenesis [36], while green tea consumption promotes lower total and LDL-cholesterol [37], and possesses anti-inflammatory properties [38]. When considering all these potential mechanisms of action and beneficial health effects of green tea consumption, it is intriguing that consumption of green tea is associated with the higher SAI. A relatively recent systematic review of the effects of green tea catechins on cardiovascular disease risk factors in human clinical trials suggests that BMI, hypertension, and plasma lipids may be improved, but several methodological issues limit the generalizability of the current evidence [6]. 

Although observed results relating to the differences between black and green tea are inconsistent, these observational associations could be potentially supported by substantial evidence from experimental studies investigating the antioxidant and vasodilation effects of the various tea compounds such as the l-theanine (l-THE) [1,2,39]. The l-THE is non-proteinous, and the most predominant amino acid found in green tea, accounting for over 50% of the total amino acid content. Concurrent with increasing commercial availability of the relatively pure l-THE [39], dose-dependent clinical trials have identified its hypotensive properties and negation of caffeine-induced effects related to increases in blood pressure [2]. Tea processing such as withering and fermentation are commonly applied to different tea varieties and only marginally influences the caffeine content [40], suggesting the importance of l-THE in the prevention of caffeine-related side effects. Green tea also contains gamma-aminobutyric acid (GABA) which in animal models has been shown to decrease blood pressure [41] and improve antioxidative defenses and mood status [42] through the modulations of neurotransmitters responsible for antidepressant-like effects [43]. Besides the content of l-THE and GABA, as well as many other unidentified potentially biologically active compounds present in tea [44], there are several other proposed mechanisms of action related to green tea consumption such as the suppression of the NADPH oxidase activity and reduction of reactive oxygen species in the vascular system [45]. Green tea polyphenols also possess anti-inflammatory properties *in vitro* through blocking of NFκB activation [46], and in animal models has been shown to decrease production of IL-6, TNFα, serum amyloid A [47] and S100β [48]. 

The positive results associated with green tea are potentially explained by the differences in the compositions of the two different tea varieties. It is well established that green tea contains higher levels of catechins in comparison to black tea, primarily due to the processing of tea leaves post-harvest [11,17]. Therefore, these higher levels of green tea catechins could be the driving factor for the lower levels of hypertension in this population sample, and similar findings have been reported in different population settings. For example, in Taiwanese men and women [49], habitual intake of green or oolong tea over 120 mL/day was associated with a reduced risk of developing hypertension. A recent systematic literature review with meta-analysis analyzed studies where green tea extracts high in catechins reduce systolic blood pressure, and total and LDL cholesterol levels [45]. Consumption of both tea varieties (black and green) can be beneficial in lowering blood pressure in individuals with prehypertensive and hypertensive ranges [50,51]. On the other hand, acute consumption of caffeine found in green and black tea may increase blood pressure, in participants who avoided caffeine for at least 12 h [52,53]. However, the relevance of these acute trials to regular tea consumption over the long term are still uncertain. Interestingly, short-term controlled clinical trials of regular tea consumption up to 8 weeks in normotensive individuals have shown that regular tea consumption does not have an overall effect on systolic or diastolic blood pressures [54,55]. Nevertheless, it is plausible that longer-term habitual consumption of tea can positively affect vasodilatory function and consequently results in altered vascular tone and blood pressure [56]. 

The population sample in the present study that consumed higher levels of green tea presented with fewer cases of hypertension but were also more physically active and also more likely to be male. Taking into consideration that SAI is constructed using ten attributes including the physical activity, BMI, depression, cardiovascular disease risk factors, as well as adherence to Mediterranean diet [57], it is intriguing that green tea consumption has shown higher odds for having SAI if compared to those of drinking black tea. Both groups consuming black and green tea had greater adherence to a Mediterranean diet pattern compared with the overall sample. Therefore, the divergent results for each type of tea were not confounded by the overall dietary pattern. In addition, prevalence of hypercholesterolemia and other cardiometabolic risk factors were not different between the types of tea. As such, it is plausible that green tea could be considered as a proxy for an overall healthier lifestyle in this population, and the results may be confounded by healthy user bias in green tea consumers, leading to a higher rating of the SAI. In particular, physical activity represents a potential confounding variable as it indicates green tea drinkers may be more health conscious, however, the green tea group also contained more smokers compared with black tea group, although the difference was not statistically significant. 

Importantly, when proposing potential beneficial health effects of tea (and tea beverages) the quantity and the stability of the tea catechins (and other compounds) during the consumption and preparation of the beverages must be considered. From this perspective, temperature and exposure time (brewing time) pose an essential role in the stability of the tea components [58] as much as the quality of the water used in the preparation of a tea [59] and the repeated use of water during the tea preparation [60]. The latter is more prevalent (and somewhat exclusive) in Asian societies, making it very hard to count accurately the number of cups as a delivery method of tea flavanols (catechins) as the measure of exposure.

To our knowledge, this is one of the first studies to investigate the consumption of green and black tea, and tea overall, in the Mediterranean region in relation to the successful ageing of the older adults. Two large population-based samples were analyzed in combination, reducing the potential for bias and increasing the external validity of the findings. A strength of the study is also the adjustment for confounding based on coffee consumption. The effects of coffee on overall health are relatively controversial but have been associated with lower all-cause mortality [61]. Nevertheless, this study has several limitations. Dietary habits and participants data were only measured through one food frequency questionnaires (FFQ) with potential for bias towards an inaccurate recall of food intake or tendency to over-report ‘*healthy*’ foods such as green tea. The FFQ could not evaluate the main catechins, polymerized polyphenols, and amino acid compositions in the observed teas. Therefore, the direct effects of these constituents cannot be readily established. However, adherence to a Mediterranean style dietary pattern was similar in both green and black tea drinkers, strengthening the findings of benefits due to green tea consumption. Another limitation of the analysis is both consumers of green and black tea scored lower on the SAI than the overall population sample which may be related to the higher age of both tea drinking groups. Higher percentages of hypertension, diabetes, and hypercholesterolemia were also observed in the tea drinking groups, which may also be contributing to the lower SAI score compared with the overall sample. 

In conclusion, the consumption of green tea was associated with a higher likelihood of successful aging in a population sample in Greece. Interestingly, consumption of black tea was negatively associated with successful ageing and potential reasons for this observation definitely raise several questions related to the chemical composition of the two different tea varieties. The positive effects of green tea may be associated with their higher levels of catechins and other predominant constituents such as l-THE, GABA and other biologically active compounds. However, green tea consumption was also associated with higher levels of physical activity and lower prevalence of hypertension, suggesting green tea is consumed by individuals living an overall healthier lifestyle in the region. 

## 4. Materials and Methods 

The study sample consisted of participants of the ATTICA and MEDIS studies, aged > 50 years old, residing in urban and insular Greek areas. As previously detailed [62], the ATTICA study was a population-based observational study implemented in the greater metropolitan Athens area, during 2001–2002. At baseline, all participants were free of CVD and cancer, as assessed through a detailed clinical evaluation by the study’s physicians. From the original sample (*n* = 2583) over 18 years old, of the ATTICA sample, a sub-group of *n* = 1128 individuals aged > 50 years old were analyzed for the purposes of the present work. In addition, as previously detailed [57], based on the MEDIS study, approximately 3000 older people from Mani (Greek peninsula region) and 26 Mediterranean islands of 5 countries were enrolled during 2005–2017 (MEDIS study). Individuals who resided in assisted-living centers, had a clinical history of cardiovascular disease (CVD) or cancer, or had left the island for a considerable period of time during their life (i.e., >5 years) were excluded. Of the 3138 MEDIS study participants aged > 50 years living in the insular Mediterranean region (Cyprus, Spain, Italy, Turkey and Greece), a sub-group of *n* = 2221 individuals from Greek islands were analyzed in this work. For both aforementioned studies, a group of trained health scientists (including cardiologists, general practitioners, physicians, dietitians, public health nutritionists, and nurses) collected all information using standard, validated questionnaires and clinical procedures. 

### 4.1. Bioethics

The ATTICA study was approved by the Bioethics Committee of Athens Medical School and was carried out in accordance with the Declaration of Helsinki (1989) of the World Medical Association. The MEDIS study was approved by the Institutional Ethics Board of Harokopio University (16/19-12-2006) and followed the ethical recommendations of the World Medical Association (52nd WMA General Assembly, Edinburgh, Scotland, October 2000). In both studies, participants were informed of the study aims and procedures, and provided written informed consent for study participation prior to enrolment.

### 4.2. Measurements

#### 4.2.1. Sociodemographic Data

The sociodemographic characteristics assessed within the context of the present investigation were age (years), gender (male/female), and smoking status. Current smokers were defined as those who smoked at least one cigarette or any type of tobacco per day at the time of the interview. Former smokers were defined as those who previously smoked but had quit within the previous year. Current and former smokers were combined as ever smokers. The remaining participants were defined as non-smokers.

#### 4.2.2. Physical Activity Levels

Physical activity was evaluated in Metabolic equivalent (MET) minutes per week, using the shortened, translated and validated in Greek version of the self-reported International Physical Activity Questionnaire (IPAQ) [62]. Those who reported at least 3 MET-minutes per week were classified as physically active. All others were defined as physically inactive.

#### 4.2.3. Anthropometric and Clinical Characteristics

Weight and height were measured using standard procedures to attain volunteer’s Body Mass Index (BMI) (kg/m^2^). Overweight was defined as BMI between 25.0 and 29.9 kg/m^2^, while obesity was defined as BMI > 29.9 kg/m^2^. Type 2 diabetes mellitus (T2DM) was determined by measuring fasting plasma glucose and in accordance with the American Diabetes Association diagnostic criteria (fasting blood glucose > 126 mg/dL or use of antidiabetic medication). Participants who had blood pressure levels > 140/90 mm Hg or who were administered antihypertensive medications were classified as hypertensive. Fasting blood lipids levels were also recorded. Hypercholesterolemia was defined as total serum cholesterol levels > 200 mg/dL or the use of lipid-lowering agents according to the National Cholesterol Education Program Adult Treatment Panel III guidelines [63]. The coefficient of variation for the blood measurements was less than 5%. A cumulative indicator of Cardiometabolic Risk, indicating the overall burden of known cardiometabolic risk factors (i.e., obesity and history of hypertension, T2DM, and hypercholesterolemia) was constructed (score range 0–4), wherein participants having none of the aforementioned risk factors were assigned a score of 0, having one factor a score of 1, etc.

#### 4.2.4. Dietary Habits Assessment

Among ATTICA study participants, the evaluation of dietary habits was based on a semi-quantitative food-frequency questionnaire (FFQ), originally developed for the European Prospective Investigation into Cancer and Nutrition (EPIC) study [64]. The Greek version of the EPIC questionnaire was provided by the Unit of Nutrition of Athens Medical School, after being translated according to standard literature guidelines [65]. Participants were requested to report the average intake (per week or per day) of several food items that they have been consuming (during the last 12 months). Similar to the ATTICA study, dietary habits in the MEDIS study were assessed through a semi-quantitative, validated, and reproducible FFQ [66]. In both studies, consumption of various food groups and beverages (i.e., meat and meat products, poultry, fish, milk and other dairy products, fruits, vegetables, greens and salads, legumes, cereals, pasta, soft drinks, coffee and tea) was measured, in times of weekly consumption (never, rare, 2–3 times/month, 1–2 times/week, 3–5 times per week and daily). Particularly for tea consumption all participants were asked about the type of tea (green/black) and the frequency they consume a cup (of 150 mL) within a week (i.e., never, <1 cup/week, 1–2 cups/day, 3–5 cups/day, >5 cups/day. Moreover, overall assessment of dietary habits was evaluated through MedDietScore (range 0–55) that assesses adherence to the Mediterranean dietary pattern [67].

#### 4.2.5. Successful Aging Index

A Successful Aging Index (SAI), ranging from 0 to 10, which has been previously developed and validated [57], using 10 attributes that reflect and have been found associated with the aging process, was applied for assessing successful ageing. The index encompasses health-related social-, lifestyle- and clinical factors, including education, financial status, physical activity, BMI, depression, participation in social activities with friends and family, number of yearly excursions, total number of clinical CVD risk factors (i.e., history of hypertension, diabetes, hypercholesterolemia, obesity) and level of adherence to the Mediterranean diet. Specifically: Education (years of school): 0–2 years = 0, 3–6 years = 0.33, 7–12 years = 0.66, >12 years = 1, Financial status (self-reported): Low = 0, moderate = 0.33, good = 0.66, very good = 1, Social activities with friends (per week): None = 0, 1 time = 0.25, 2 times = 0.5, 3–5 times = 0.75, > 5 times = 1, Social activities with family (per week): None = 0, 1 time = 0.25, 2 times = 0.5, 3–5 times = 0.75, > 5 times = 1, Going to excursions: None = 0, 1 time = 0.25, 2 times = 0.5, 3–5 times = 0.75, > 5 times = 1, Physical activity (per week): None = 0, 1–2 times = 0.33, 3–5 times = 0.66, >5 times = 1, BMI classes: Normal weight: 1, overweight = 0.5, underweight or obese = 0, Geriatric Depression Scale (GDS) (0–15): 0–4 points (no depression) = 1, 5–10 points (mild depression) = 0.5, >10 points (severe depression) = 0, CVD risk score (i.e., obesity, history of hypertension, diabetes, hypercholesterolemia): None = 1, 1 factor = 0.75, 2 factors = 0.5, 3 factors = 0.25, 4 factors = 0, and Level of adherence to the Mediterranean diet (MedDietScore 0–55): 0–34 points = 0, 35–38 points = 0.5, > 38 points = 1.

### 4.3. Statistical Analysis

Continuous variables are presented as mean ± standard deviation (SD, and categorical variables as frequencies. Associations between continuous variables and group of participants were evaluated with Student’s *t*-test; Spearman rho correlation coefficient was used to evaluate relationships between cups of tea drinking and SAI score. Multiple linear regression models were used to evaluate the associations between tea consumption (independent variables), adjusted for various participants’ characteristics (i.e., age, gender, coffee consumption and smoking habits) and the SAI (dependent outcome). The Results are presented as unstandardized beta coefficients ± standard error (b ± SE) and *p*-value. Linearity of models’ fitting was tested through the scatter plots of standardized residuals against fitted values; normality of regression residuals was evaluated through P-P plots; dependency was tested using the Durbin-Watson test and homoscedacity using the Variance Inflation Index (value <4 suggests lack of heteroscedacity). Moreover, multiple binary logistic regression models were used to evaluate the association between the type of tea (Green vs Black tea, Green vs Black or no tea at all [control group], and Black vs Green or control group) (independent variables) with the level of successful aging index (i.e., above or below the median value 3.58) (dependent variable). The results are presented as Odds Ratios (OR) of the likelihood of having SAI above the median value and their 95% confidence intervals. Deviance residuals were used to evaluate models’ goodness-of-fit. Tea consumption was assessed as “drinking” or “not-drinking” (i.e., yes/no) in all multi-adjusted analyses because of lack adequate data in each consumption category. STATA (College Station, TX, USA & M. Psarros et Assoc., Sparta, Greece) software version 13 was used for all calculations.

## Figures and Tables

**Table 1:** Sociodemographic, lifestyle, and clinical characteristics of the ATTICA and MEDIS study participants based on the type of tea (green/black) they consume.

	MEDIS and ATTICA Study Participants * (*n* = 3349)	Green Tea Consumers (*n* = 595)	Black Tea Consumers (*n* = 600)	*p*
Age (years)	69 ± 10	74 ± 8.3	74 ± 7.0	0.300
Male n (%)	1751 (52)	318 (53)	281 (47)	0.026
Female n (%)	1598 (48)	277 (47)	319 (53)	0.026
Ever smoking n (%)	1358 (43)	217 (39)	198 (34)	0.111
Physically active n (%)	1372 (41)	278 (47)	219 (37)	<0.001
BMI (kg/m^2^)	28 ± 4.4	29 ± 4.7	29 ± 4.8	0.129
Hypertension n (%yes)	1881 (86)	362 (89)	423 (94)	0.006
Diabetes n (%yes)	696 (21)	146 (25)	139 (23)	0.578
Hypercholesterolemia n (%yes)	1747 (53)	327 (55)	317 (53)	0.458
Coffee n (% yes)	2791 (85)	525 (88)	548 (92)	0.087
*Frequency of tea consumption*				
0–1 cup	796 (67)	390 (66)	406 (73)	<0.001
1–2 cups	238 (20)	124 (21)	114 (18)	<0.001
3–5 cups	124 (10)	68 (11)	56 (8)	<0.001
5+ cups	34 (3)	14 (2)	20 (1)	0.001
MedDietScore (0–55)	29 ± 7.1	33 ± 4.6	33 ± 4.9	0.868
Cardiometabolic risk factors (0–4)	1.6 ± 1.1	1.7 ± 1.0	1.9 ± 1.1	0.092
SAI (0–10)	3.1 ± 1.2	2.9 ± 1.4	2.3 ± 1.3	<0.001

* All MEDIS and ATTICA study participants consuming or not tea, or without information about tea consumption. Values are presented as percent (%) or mean ± standard deviation. *p*: probability values between green tea and black tea consumers derived from Student’s *t*-test for continuous variables or the chi-square test for the categorical variables.

**Table 2:** Results from five linear regression models that evaluated the association between the type (Green or Black) of tea consumed and successful aging (dependent outcome), after adjusting for age, sex, smoking and coffee consumption, among ATTICA and MEDIS study participants.

	b ± SE	*p*
Model 1 adj. for age, sex, smoking and coffee consumption + Green vs. Black tea	0.503 ± 0.078	<0.001
Model 2 adj. for age, sex, smoking and coffee consumption + Green vs. Black tea or Control group	0.020 ± 0.057	0.722
Model 3 adj. for age, sex, smoking and coffee consumption + Green tea vs. Control group	0.225 ± 0.055	<0.001
Model 4 adj. for age, sex, smoking and coffee consumption + Black vs. Green tea or Control group	−0.727 ± 0.055	<0.001
Model 5 adj. for age, sex, smoking and coffee consumption + Black tea vs. Control group	−0.807 ± 0.054	<0.001

Results for the association of tea consumption (yes/no) on SAI (i.e., the higher the better) are presented as unstandardized b coefficients ± Standard Error and *p*-value. Control group: no tea drinking.

**Table 3:** Results from logistic regression models that evaluated the association between the type (Green or Black) of tea consumed with successful aging index (i.e., above or below the median value 3.58) (dependent variable) among ATTICA and MEDIS study participants, separately for males and females.

	OR	95% CI	*p*
**All Participants**			
Model 1 adj. for age, sex, smoking and coffee consumption + Green vs. Black tea	1.77	1.38–2.28	<0.001
Model 2 adj. for age, sex, smoking and coffee consumption + Green vs. Black tea or Control group	1.41	1.15–1.73	0.001
Model 3 adj. for age, sex, smoking and coffee consumption + Green tea vs. Control group	1.20	1.05–1.51	0.048
Model 4 adj. for age, sex, smoking and coffee consumption + Black vs. Green tea or Control group	0.62	0.51–0.77	<0.001
Model 5 adj. for age, sex, smoking and coffee consumption + Black tea vs. Control group	0.69	0.55–0.86	0.001
**Females**			
Model 1 adj. for age, sex, smoking and coffee consumption + Green vs. Black tea	1.75	1.22–2.52	0.003
Model 2 adj. for age, sex, smoking and coffee consumption + Green vs. Black tea or Control group	1.36	1.01–1.84	0.049
Model 3 adj. for age, sex, smoking and coffee consumption + Green tea vs. Control group	1.12	0.80–1.56	0.527
Model 4 adj. for age, sex, smoking and coffee consumption + Black vs. Green tea or Control group	0.62	0.46–0.83	0.002
Model 5 adj. for age, sex, smoking and coffee consumption + Black tea vs. Control group	0.63	0.45–0.89	0.07
**Males**			
Model 1 adj. for age, sex, smoking and coffee consumption + Green vs. Black tea	1.79	1.26–2.53	0.001
Model 2 adj. for age, sex, smoking and coffee consumption + Green vs. Black tea or Control group	1.50	1.12–2.01	0.007
Model 3 adj. for age, sex, smoking and coffee consumption + Green tea vs. Control group	1.31	0.96–1.80	0.093
Model 4 adj. for age, sex, smoking and coffee consumption + Black vs. Green tea or Control group	0.67	0.50–0.89	0.006
Model 5 adj. for age, sex, smoking and coffee consumption + Black tea vs. Control group	0.76	0.56–1.04	0.087

Results of tea consumption (yes/no) on the likelihood of having higher SAI (i.e., better aging) are presented as Odds Ratios (OR), their corresponding 95% CI and *p*-value.
